# Suboptimal reliability of FIB‐4 and NAFLD‐fibrosis scores for staging of liver fibrosis in general population

**DOI:** 10.1002/jgh3.13034

**Published:** 2024-02-20

**Authors:** Rawi Hazzan, Nur Abu Ahmad, Abu Shqara Habib, Igbaria Saleh, Neeman Ziv

**Affiliations:** ^1^ Liver Clinic Emek Medical Center Afula Israel; ^2^ Diagnostic Imaging Institute Emek Medical Center Afula Israel; ^3^ The Faculty of Medicine Technion Institute of Technology Haifa Israel

**Keywords:** liver cirrhosis, noninvasive methods, shear wave elastography, transient elastography

## Abstract

**Background and Aim:**

The burden and incidence of liver cirrhosis are increasing worldwide. Early detection of liver fibrosis would help in early interventions and preventing the progression of fibrosis and cirrhosis. The accepted noninvasive markers for liver fibrosis staging, namely fibrosis‐4 (FIB‐4) and nonalcoholic fatty liver disease fibrosis score (NFS), have shown inconsistent performance for detecting the fibrosis stage. We aimed to evaluate the efficacy of FIB‐4 score and NFS for the detection of liver fibrosis in the general population.

**Methods:**

From a general population referred from a single, community‐based family‐physician clinic, we included study participants between the ages of 45 and 65 years, with no knowledge of liver disease and no record of alcohol consumption. Liver fibrosis was evaluated by the FIB‐4 score and NFS using shear wave elastography (SWE) or transient elastography (TE) measurements as a reference.

**Results:**

A total of 76 participants (aged 61.5 ± 0.37 years, 33% females) were included in the study cohort. We observed a nonsignificant correlation between liver stiffness measurement (LSM) and FIB‐4 and NFS (*r* = 0.1, *P* = 0.37; *r* = 0.16, *P* = 0.15, respectively). Our results showed that only 5.2% with FIB‐4 >3.25 and 9.7% with NFS >0.675 had LSM >12 kPa. The compatibility of fibrosis staging was 55% between FIB‐4 score and LSM and only 18% between NFS and LSM.

**Conclusion:**

We found that FIB‐4 and NFS are unreliable tools for liver fibrosis estimation in the general population. There is a need for more reliable noninvasive methods for the early detection of liver fibrosis.

## Graphical abstract

A total of 76 participants were included in the study cohort. We observed a nonsignificant correlation between liver stiffness measurement (LSM) and FIB‐4 and NFS (r=0.1, P=0.37; r=0.16, P=0.15, respectively). The results showed that only 5.2% with FIB‐4 >3.25 and 9.7% with NFS >0.675 had LSM >12kPa. The compatibility of fibrosis staging was 55% between FIB‐4 score and LSM and only 18% between NFS and LSM. FIB‐4 and NFS are unreliable tools for liver fibrosis estimation in the general population.

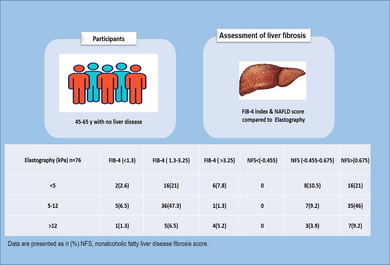

## Introduction

The burden and incidence of liver disease are increasing worldwide. Nonalcoholic fatty liver disease (NAFLD) is the most common etiology for chronic liver disease, with a prevalence of ~35% worldwide.^1^ NAFLD can be defined as the presence of fatty deposits in the liver, which has the potential to develop into nonalcoholic steatohepatitis (NASH), a more advanced and serious stage of the disease. NASH consists of liver inflammation and hepatocyte injury, which could develop into liver fibrosis and further into liver cirrhosis and liver cancer^2^

Liver cirrhosis ranks 11th in terms of frequency and is among the top 10 leading causes of death. Its incidence is steadily rising.^3^ Therefore, there is an urgent need to classify asymptomatic patients with liver diseases in the early stages to determine patient care needs and inhibit/prevent progression of liver fibrosis.

Liver biopsy is the gold standard for the diagnosis of liver fibrosis or liver cirrhosis.^4^ However, liver biopsy is an invasive and complicated procedure for the patients and is associated with sampling mistakes and not free of risks, and thus not practical and possibly unethical in asymptomatic cases.^5^ Moreover, biopsy sample length affects the accuracy of fibrosis assessment; uneven distribution of the fibrotic process and variable assessments can lead to altered test result, bleeding, and death.^6^ So, several studies have considered the evaluation of noninvasive methods for the diagnosis of liver fibrosis stages.

The combination of liver fibrosis biomarkers, such as fibrosis‐4 (FIB‐4) and nonalcoholic fatty liver disease fibrosis score (NFS), has been extensively studied as noninvasive and acceptable screening tests for excluding advanced fibrosis. In a prospective study involving 81 patients with biopsy‐proven advanced fibrosis, the FIB‐4 score and NFS effectively ruled out advanced fibrosis, resulting in a 67% reduction in the necessity for biopsies.^7^ Similarly, retrospective studies on patients with NAFLD and metabolic‐associated fatty liver disease (MAFLD) have shown that FIB‐4 and NFS classify individuals as low risk for advanced fibrosis.^8^, ^9^

Furthermore, Thévenot *et al*. investigated noninvasive tests (NITs) for screening advanced liver fibrosis in 189 patients with coronary artery disease and NAFLD. Their findings suggested that NFS or the easy liver fibrosis test (eLIFT) were the most promising first‐line biochemical NITs for identifying suitable candidates for Fibroscan.^10^ However, Graupera *et al*. found that in patients at high risk of liver disease, FIB‐4 and NFS are inadequate tools for screening liver fibrosis staging.^11^

In the last decade, liver fibrosis has also been detected by imaging methods such as ultrasound elastography.^12^ Transient elastography (TE) and shear wave elastography (SWE) are well‐established noninvasive ultrasound‐based techniques for the staging and detection of fibrosis in patients with liver disease of different etiologies.^13^, ^14^ However, both methods have limitations, such as the need for an experienced sonographer, as well as the relatively low accessibility of these imaging methods in community medicine.^15^, ^16^

Because early stages of liver fibrosis and cirrhosis could be reversible, and because the findings regarding the prediction accuracy for liver fibrosis of both FIB‐4 and NFS are inconsistent, we aimed to determine the efficacy of FIB‐4 and NFS for fibrosis staging in asymptomatic patients, using TE and SWE as reference methods. Liver stiffness measurement (LSM) >12 kPa was used as a cutoff for the detection of liver cirrhosis.^17^

## Methods

**Figure 1:**
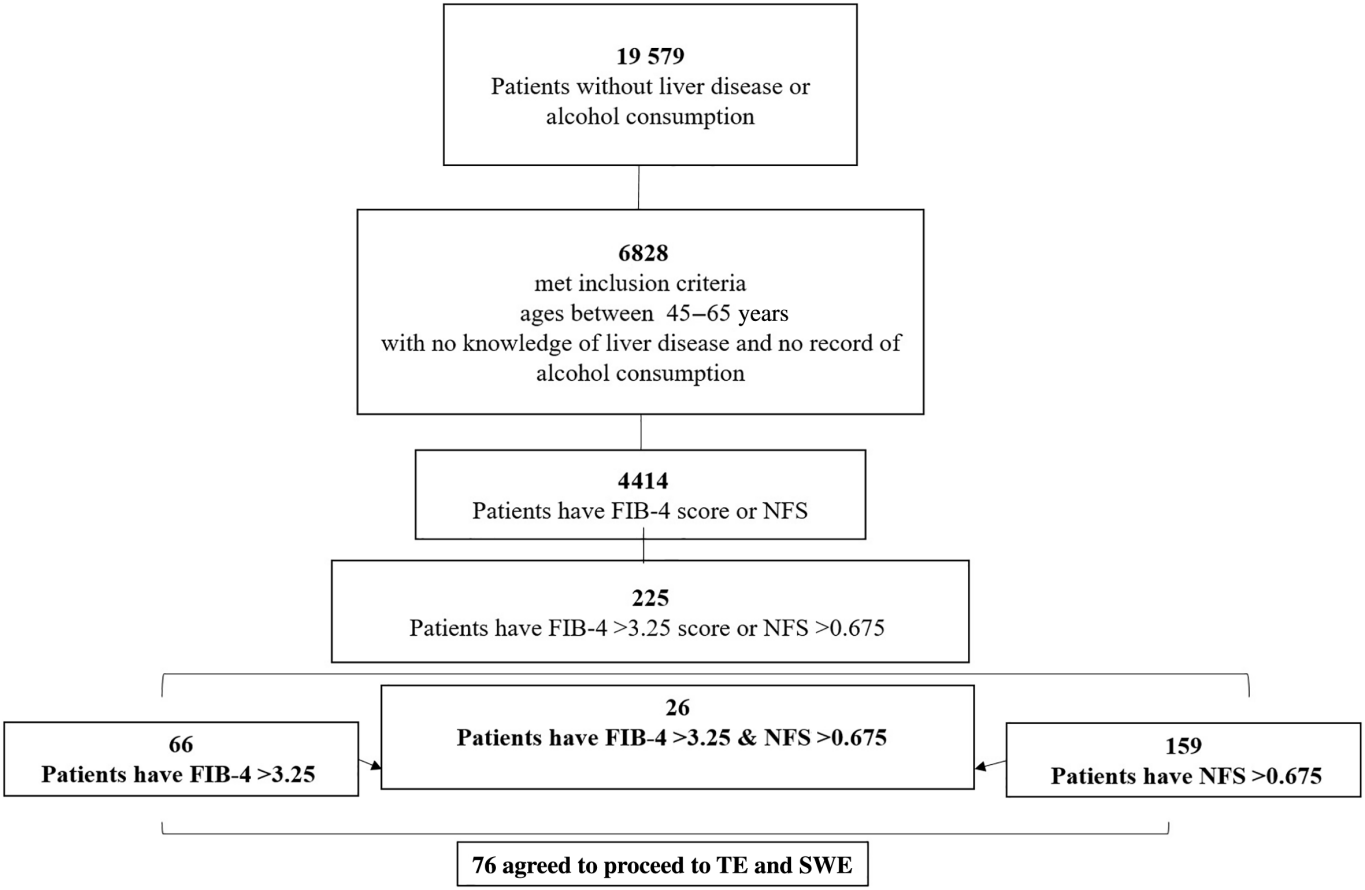
Experimental design of the study. FIB‐4, fibrosis‐4; NFS, nonalcoholic fatty liver disease fibrosis score; SWE, shear wave elastography; TE, transient elastography.

From a general population of 19 579 patients referred from a single, community‐based family‐physician clinic, we included only study participants between the ages of 45 and 65 years, with no knowledge of liver disease and no record of alcohol consumption. Study participants with positive serology for hepatitis B virus (HBV) and C (HCV) and evidence for right heart failure were excluded from the study. Figure 1 shows the steps of the enrollment process of the participants. The study was approved by the Helsinki Committee, No. 0007‐18‐EMC, on 7 June 2018.

Clinical evaluation of the study participants was carried out based on the participants' medical records.

Liver fibrosis was evaluated by two noninvasive methods (FIB‐4 and NFS) using SWE or TE as a reference method. Table 1 presents cut‐off points for all methods that were used for fibrosis grading. Liver fibrosis risk was classified into three stages: F0–F1, low risk; F2–F3, indeterminate risk; F4, high risk for cirrhosis.

**Table 1:** Cut‐off points for all methods that were used for fibrosis staging

Method	FIB‐4 score	NFS	Elastography (kPa)
Low risk of advanced fibrosis	<1.3	<−1.455	<5
Indeterminate risk of advanced fibrosis	1.3–3.25	−1.455 to 0.675	5–12
High risk of advanced fibrosis	>3.25	>0.675	>12

FIB‐4, fibrosis‐4; NFS, nonalcoholic fatty liver disease fibrosis score.

### Liver stiffness measurements by elastography

LS (kPa) was performed using SWE or TE (SuperSonic Imagine, Aix‐en‐Provence, France). SWE or TE was conducted for all participants by a skilled sonographer. All patients were fasted for 8 h and were asked to stop drinking alcohol and exercising for 12 h and 20 min, respectively, prior to the examination. Measurements were performed in a supine position with the right arm elevated. Scans were performed after a small breath‐hold, using an intercostal approach, with sampling >1 cm from the liver surface and away from major vessels. Two measurements were obtained from the right lobe of the liver. Images were interpreted by a single, blinded senior radiologist.

### Statistical analysis

#### Sample size

Comparison between FIB‐4 score and elastography for advanced fibrosis was of primary interest, and the sample size was determined for the difference between FIB‐4 and elastography for advanced liver fibrosis. According to preliminary testing in a pilot study of 13 participants, which was performed to identify any unexpected problems, we observed that the proportion of advanced fibrosis was 60% and 80%, as estimated by FIB‐4 and elastography, respectively. In order to validate this hypothesis, the study requires an allocation of 82 patients. However, the present study included only 76 participants as a result of dropouts. A nonparametric McNemar test on such a sample size will achieve a power of 80% at a significance level of 5%.^18^

All statistical tests were performed with the statistical software GraphPad Prism 9.0 (GraphPad Software, San Diego, CA, USA). Baseline characteristics of participants are presented as means ± standard error of the mean for continuous variables and as percentages for categorical variables.

The Pearson correlation coefficient was used to examine correlations between FIB‐4 or NFS and LSM by elastography.

## Results

### Demographics and background characteristics of participants

Out of the 6828 participants who met the inclusion criteria, 225 had FIB‐4 and NFS evaluations. Our study included 76 participants who agreed to undergo SWE or TE. Table 2 describes the baseline characteristics of participants; the mean age was 61.5 ± 0.37 years, and 33% of participants were females. Obesity (BMI ≥ 30) was present in 63.6% of the participants (32 ± 0.6), and type‐2 diabetes was present in 67.5%. Mean aspartate aminotransferase (AST) and alanine transaminase (ALT) were 30 ± 2.9 and 24.4 ± 1.7 U/L, respectively (Table 2).

**Table 2:** Demographics and background characteristics

Parameters (*n* = 76)	Results
Age (years)	61.5 (0.37)
Sex, female	33 (43%)
BMI (kg/m^2^)	32 (0.6)
Non‐obese (BMI < 30)	28 (36.4)
Obese (BMI ≥ 30)	49 (63.6)
Diabetes	52 (67.5)
AST (U/L)	30 (2.9)
ALT (U/L)	24.4 (1.7)
Platelets (10^−9^/L)	165.4 (5.1)
Glucose (mg/dL)	123 (4.6)
HbA1C (*n* = 39)	6.4 (0.1)
Albumin (mg/dL)	4 (0.04)
FIB‐4 score	2.3 (0.1)
NFS	0.9 (0.07)
TE or SWE (kPa)	8.7 (1.1)
CAP (*n* = 53)	288 (10.5)

Data are presented as mean (standard error of the mean) or *n* (%).

ALT, alanine transaminase; AST, aspartate transaminase; BMI, body mass index; CAP, controlled attenuation parameter; FIB‐4, fibrosis‐4; Hb, hemoglobin; NFS, nonalcoholic fatty liver disease fibrosis score; SWE, shear wave elastography; TE, transient elastography.

### Performance of FIB‐4 score and NFS compared to LSM

**Figure 2:**
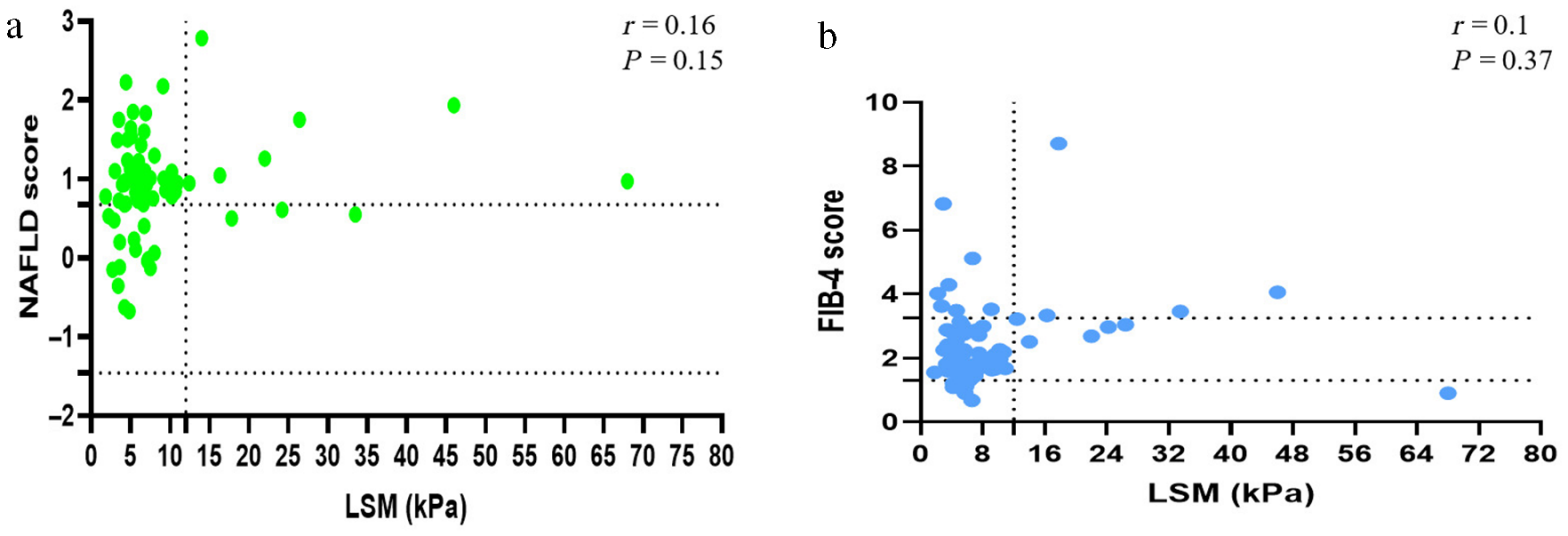
Correlation between fibrosis scores by liver stiffness measurement (LSM) (kPa) and (a) nonalcoholic fatty liver disease fibrosis score (NFS) and (b) fibrosis‐4 (FIB‐4) score. Poor correlation was observed between LSM and NFS and FIB‐4 score.NAFLD, nonalcoholic fatty liver disease.

We observed a nonsignificant correlation between LSM and FIB‐4 and NFS (*P* = 0.37, *P* = 0.15, respectively) (Fig. 2).

**Figure 3:**
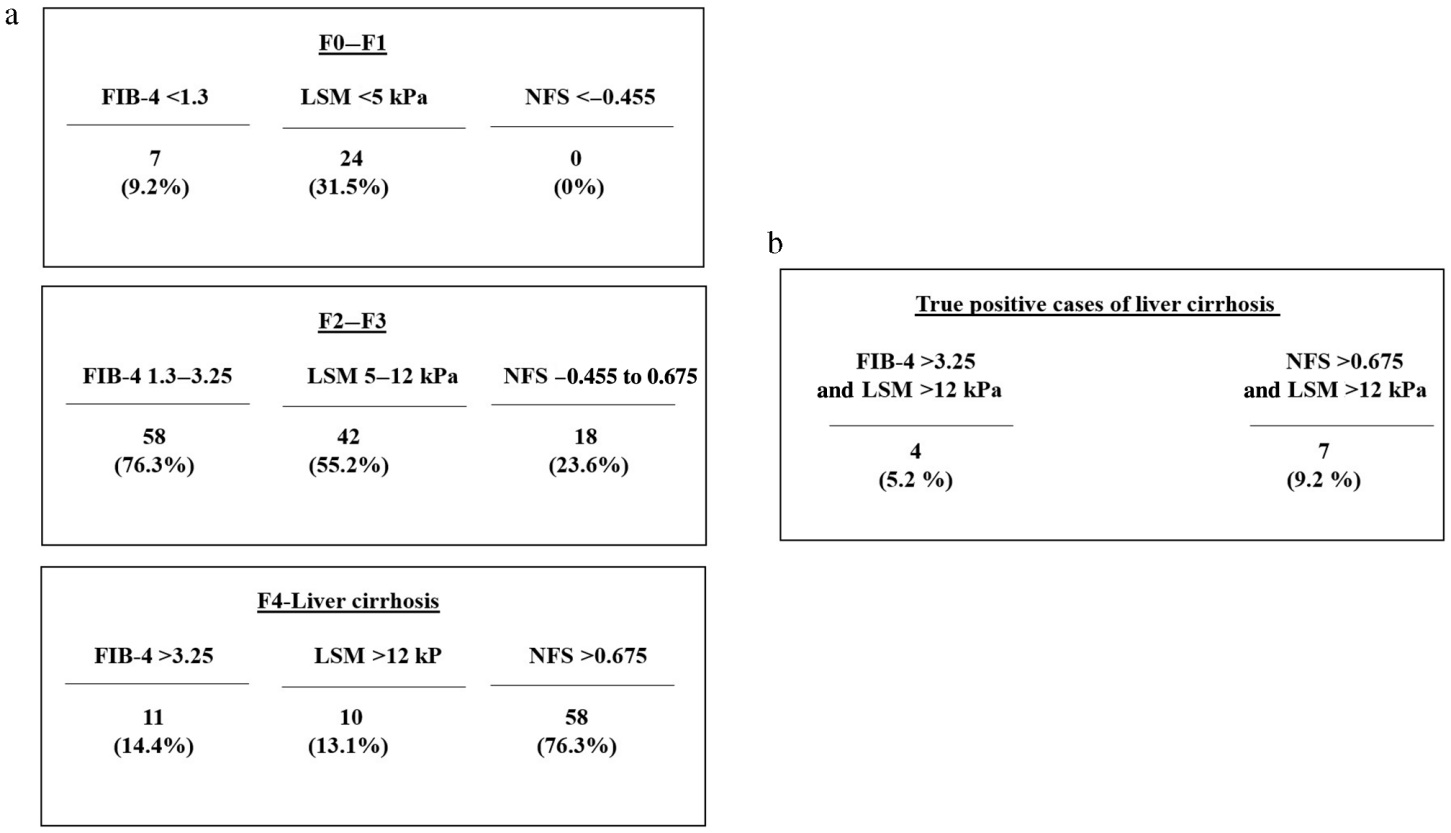
(a) Categorizing of participants by fibrosis‐4 (FIB‐4) and nonalcoholic fatty liver disease fibrosis score (NFS) and liver stiffness measurement (LSM). (b) True positive rate of liver cirrhosis classified by FIB‐4 and NFS compared with LSM in the general population.

The prevalence of LSM <5 kPa was 31.5%, while that of LSM between 5 and 12 kPa and >12 kPa was 55% and 13%, respectively. FIB‐4 <1.3, FIB‐4 between 1.3 and 3.25, and FIB‐4 >3.25 were observed in 7.9%, 76.3%, and 14.4%, respectively. All participants had NFS >−1.455, with 23.6% falling within the range −1.455 to 0.675 and 76.4% having NFS >0.675 (Fig. 3a).

Comparing the classification of fibrosis by FIB‐4 and NFS with LSM revealed that only 5.2% with FIB‐4 >3.25 and 9.7% with NFS >0.675 had LSM >12 kPa (Fig. 3b).

### Compatibility of fibrosis staging between elastography and FIB‐4 or NFS

The compatibility of fibrosis staging was 55.2% between FIB‐4 score and SWE/TE and only 18.4% between NFS and SWE/TE. The rate of false‐negative and false‐positive staging by FIB‐4 compared to LSM was 17%. However, the rate of false‐negative and false‐positive staging by NFS was 71% (Table 3).

**Table 3:** Categorizing participants based on elastography, fibrosis‐4 (FIB‐4), and nonalcoholic fatty liver disease fibrosis score (NFS)

Elastography (kPa) *n* = 76	FIB‐4 <1.3	FIB‐4 1.3–3.25	FIB‐4 >3.5	NFS <−0.455	NFS −0.455 to 0.675	NFS >0.675
<5	2 (2.6)	16 (21)	6 (7.8)	0	8 (10.5)	16 (21)
5–12	5 (6.5)	36 (47.3)	1 (1.3)	0	7 (9.2)	35 (46)
>12	1 (1.3)	5 (6.5)	4 (5.2)	0	3 (3.9)	7 (9.2)

Data are presented as *n* (%).

NFS, nonalcoholic fatty liver disease fibrosis score.

## Discussion

In the present study, we examined the efficacy of the acceptable non‐invasive methods FIB‐4 and NFS^19^, ^20^ for staging liver fibrosis in a community‐based population using SWE/TE as a reference method.^21^, ^22^ Interestingly, we found that the use of these acceptable scores (FIB‐4 and NFS) for screening for liver fibrosis may lead to incorrect evaluation of the disease.

Our findings revealed low compatibility percentage between both FIB‐4 and NFS results for liver fibrosis staging and elastography. The compatibility with elastography was 55.2% and only 18.4% with FIB‐4 and NFS, respectively, meaning that about 45% and 80% of participants would not have been identified or have been misclassified as such if the FIB‐4 score or NFS had been used alone. These results were supported by the weak correlation that we observed between elastography and both FIB‐4 and NFS. Consistently, several studies have shown the misclassification of liver fibrosis by FIB‐4 and NFS. Graupera *et al*. reported a significant percentage of false negatives and false positives with FIB‐4 and NFS in general and high risk population using TE >8 or >12 for defining significant fibrosis and cirrhosis, respectively.^11^ Also, in chronic hepatitis C patients, using FIB‐4 for fibrosis detection showed different staging results compared to fibrosis detection by TE.^23^

The findings of the present study show that FIB‐4 is better than NFS for staging liver fibrosis. The compatibility fibrosis staging of FIB‐4 (⁓55%) with elastography measurements was higher than with NFS (⁓18%). Similarly, previous studies had shown that the performance of FIB‐4 for fibrosis staging was higher than NFS score in average‐risk population.^24^ Several metabolic factors in the NFS formula could interpret the difference between FIB‐4 and NFS. Because the NFS formula uses BMI and impaired glucose tolerance, different cut‐off points of NFS may be needed for obese and diabetic participants. Indeed, about 50% of our study participants were classified as obese and diabetic, which could potentially influence the results.

Although imaging methods require specialized training and experience, they are considered the most well‐validated non‐invasive techniques for assessing liver fibrosis. Among these, SWE and TE are accepted and FDA‐approved elastographic modalities for measuring liver stiffness.^25^, ^26^ In our study, we employed SWE for assessing liver stiffness in 53 participants and TE in 23 participants because of equipment availability. Using two different imaging methods for liver fibrosis staging might introduce bias, but both SWE and TE are highly reliable for this purpose. Several studies have shown that SWE yields diagnostic performance comparable to that of TE in liver fibrosis evaluation.^27^ For instance, in a study involving 180 participants with HBV or HCV, 86.7% agreement was observed between SWE and TE results in assessing fibrosis stages.^28^ Similarly, other prospective studies have confirmed the value of both SWE and TE in staging liver fibrosis, particularly in patients with non‐NAFLD.^29^

Furthermore, prospective studies involving participants with various etiologies of chronic liver disease have concluded that SWE and TE show similar diagnostic performance at each disease stage when liver biopsy serves as the reference standard. In a recent meta‐analysis, 53 studies with TE and 12 with SWE were compared. The analysis found that TE's sensitivity, specificity, and area under the curve (AUC) results were on par with those of SWE. Specifically, the AUC for diagnosing significant fibrosis was 0.85 for TE and 0.89 for SWE, while it was similar for diagnosing cirrhosis with values of 0.89 for TE and 0.90 for SWE, respectively. It is worth noting that SWE's accuracy in detecting hepatic fibrosis was lower in patients with BMI >34.^17^ In our study, the mean BMI was 32 ± 0.6, with 36% of participants having BMI <30 and 64% having BMI >30.

Therefore, the use of both SWE and TE in the current study for the assessing fibrosis stage can be considered acceptable and is likely to yield valid results.

Additionally, in our study, when comparing the compatibility of FIB‐4 and NFS with TE and SWE separately, we observed the following consonance between these methods for liver cirrhosis staging: 5.7% for FIB‐4 and SWE (*n* = 53); 4.3% for FIB‐4 and TE (*n* = 23); 5.7% for NFS and SWE; and 17.4% for NFS and TE. When comparing elastography by SWE and TE for liver cirrhosis staging (*n* = 76), we found an agreement of 5.2% with FIB‐4 and 9.7% with NFS. These findings underscore the comparability of measurements obtained with both SWE and TE methods (Tables S1 and S2, Supporting information).

It is important to consider that employing noninvasive tests for liver fibrosis requires accounting for etiology‐specific factors and associated limitations. In our study, we included participants without a known history of liver disease and without HBV or HCV infections, suggesting the likelihood of “silent” NAFLD. Although NFS and FIB‐4 scores are widely validated for staging liver fibrosis through simple calculations, they have limitations. Notably, these tests may produce “indeterminate” range scores in at least 30% of cases and are influenced by serum biochemical abnormalities, lacking a direct assessment of liver stiffness. NFS and FIB‐4 also exhibit reduced accuracy and specificity in young (<35 years) and old (>65 years) patients, leading to the suggested age‐specific thresholds.^28^, ^30^ To address this limitation and improve the validity of these methods, we limited the age range of participants in our study to between 45 and 65 years.

In our study, we used TE and SWE as references to evaluate the effectiveness of NFS and FIB‐4 for liver fibrosis staging. Although both TE and SWE are effective for diagnosing liver fibrosis and cirrhosis, they have limitations.

TE measures liver stiffness but does not assess the extent of fibrosis, potentially leading to false‐positive results. Variable cut‐off levels in different liver diseases are another limitation, but our study mitigated this by enrolling silent NAFLD patients without HBV or HCV infection. A meta‐analysis of 1047 NAFLD patients indicates TE's high accuracy for F3 and F4 staging and moderate accuracy for F2 staging.

In the current study, we used TE and SWE as a reference to evaluate the effectiveness of NFS and FIB‐4 for the staging of liver fibrosis. Although both TE and SWE are widely recognized as effective methods for diagnosing liver fibrosis and cirrhosis, it is crucial to acknowledge certain limitations associated with these techniques. TE measures liver stiffness, but it does not assess the extent of fibrosis within the liver. This increases the potential for false‐positive results when using TE for liver fibrosis diagnosis. Additionally, the variability in cut‐off levels for diagnosing fibrosis and cirrhosis using TE in different etiologies of liver disease presents another limitation. However, as previously mentioned, our study sought to address this limitation by enrolling participants with silent NAFLD who were free from HBV or HCV infection. A meta‐analysis involving 1047 NAFLD patients suggests that the use of TE in NAFLD diagnosis provides a high degree of accuracy in identifying F3 and F4 stages and offers a moderate level of accuracy for F2 staging.^31^

SWE has similar accuracy as TE but has limitations, including susceptibility to artifacts that can significantly affect liver stiffness measurements. The requirement for specialized equipment not widely available is another constraint.^32^

Besides the limitations of these noninvasive methods, our study has several limitations, which include the small sample size; use of retrospective FIB‐4 and NFS data that might affect the consistency of results; and reliance on ultrasonography for NAFLD severity assessment, instead of liver biopsy, which is the gold standard for diagnosis.

In conclusion, FIB‐4 score and NFS are unreliable tools for liver fibrosis estimation. Our findings suggest that it would be worthwhile to identify better noninvasive, cost‐effective methods for staging liver fibrosis and cirrhosis. Future research should focus on developing and validating technically simple and practical methods for screening liver fibrosis in the general population. Combining two noninvasive methods may enhance accuracy and potentially address some of the limitations associated with single tests.

## Supporting information


**Table S1.** Demographics and background characteristics according to SWE and TE individually.

**Table S2.** Categorizing participants according to SWE and TE individually.
