# Supplementary material for: Suboptimal reliability of FIB‐4 and NAFLD‐fibrosis scores for staging of liver fibrosis in general population
Source: JGH Open. 2024 Feb 20;8(2):e13034. doi: 10.1002/jgh3.13034 (PMC10877654; doi:10.1002/jgh3.13034)
Supplement: Supplementary file 1 — Table S1. Demographics and background characteristics according to SWE and TE individually. [file JGH3-8-e13034-s002.pptx]

## Slide 1
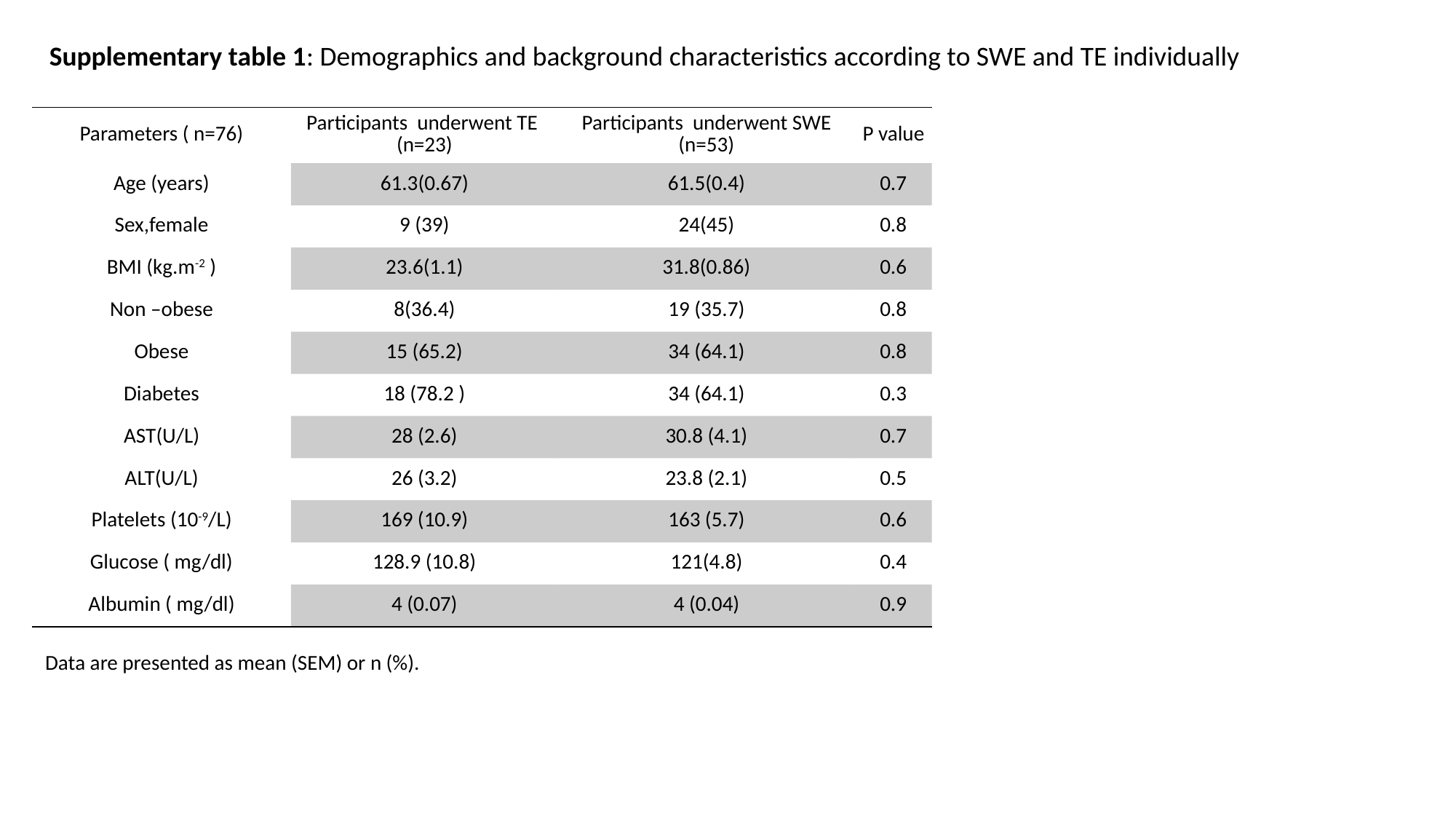

Supplementary table 1: Demographics and background characteristics according to SWE and TE individually
| Parameters ( n=76) | Participants underwent TE (n=23) | Participants underwent SWE (n=53) | P value |
| --- | --- | --- | --- |
| Age (years) | 61.3(0.67) | 61.5(0.4) | 0.7 |
| Sex,female | 9 (39) | 24(45) | 0.8 |
| BMI (kg.m-2 ) | 23.6(1.1) | 31.8(0.86) | 0.6 |
| Non –obese | 8(36.4) | 19 (35.7) | 0.8 |
| Obese | 15 (65.2) | 34 (64.1) | 0.8 |
| Diabetes | 18 (78.2 ) | 34 (64.1) | 0.3 |
| AST(U/L) | 28 (2.6) | 30.8 (4.1) | 0.7 |
| ALT(U/L) | 26 (3.2) | 23.8 (2.1) | 0.5 |
| Platelets (10-9/L) | 169 (10.9) | 163 (5.7) | 0.6 |
| Glucose ( mg/dl) | 128.9 (10.8) | 121(4.8) | 0.4 |
| Albumin ( mg/dl) | 4 (0.07) | 4 (0.04) | 0.9 |
Data are presented as mean (SEM) or n (%).
