# Supplementary material for: Suboptimal reliability of FIB‐4 and NAFLD‐fibrosis scores for staging of liver fibrosis in general population
Source: JGH Open. 2024 Feb 20;8(2):e13034. doi: 10.1002/jgh3.13034 (PMC10877654; doi:10.1002/jgh3.13034)
Supplement: Supplementary file 2 — Table S2. Categorizing participants according to SWE and TE individually. [file JGH3-8-e13034-s001.pptx]

## Slide 1
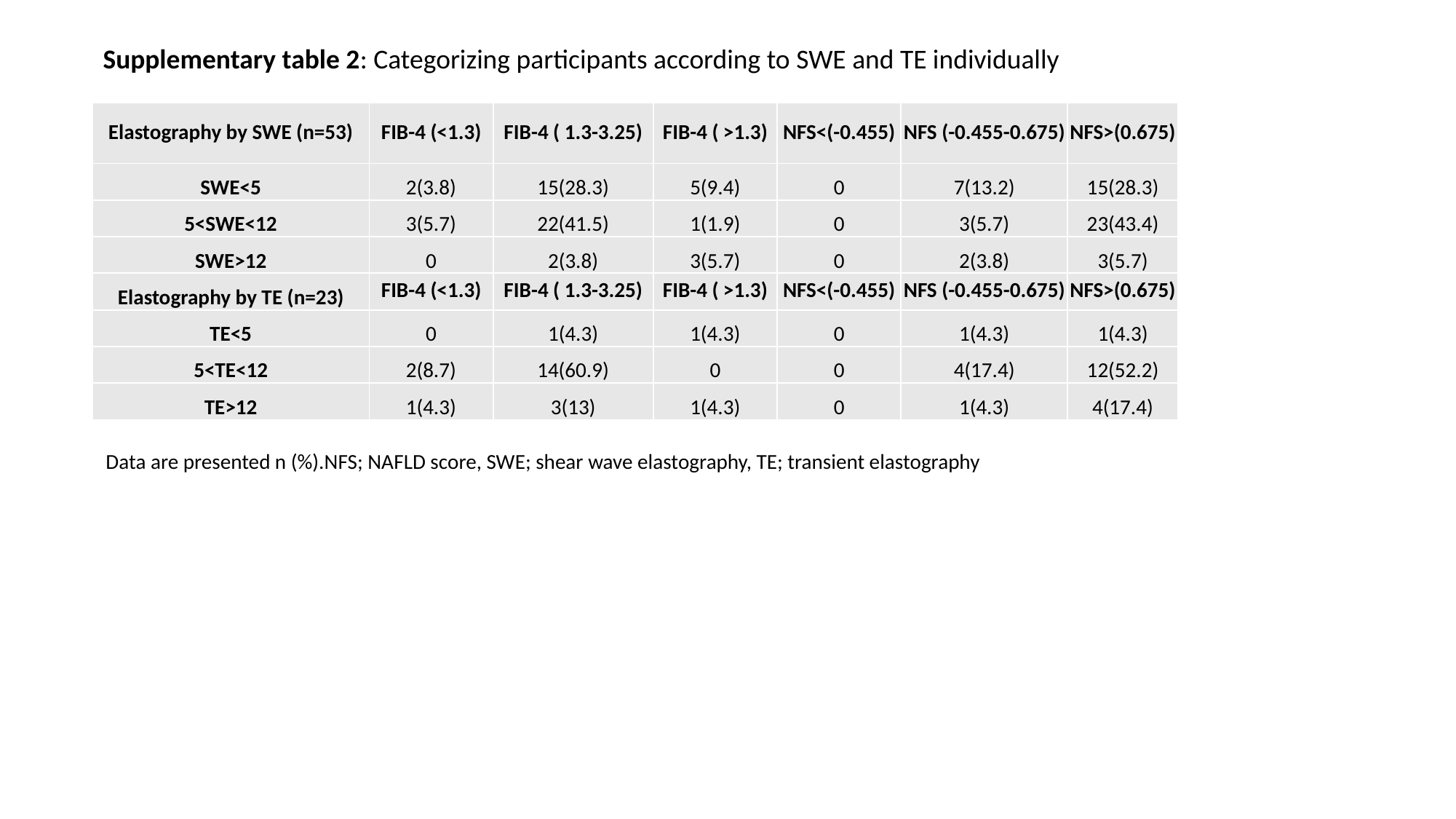

Supplementary table 2: Categorizing participants according to SWE and TE individually
| Elastography by SWE (n=53) | FIB-4 (<1.3) | FIB-4 ( 1.3-3.25) | FIB-4 ( >1.3) | NFS<(-0.455) | NFS (-0.455-0.675) | NFS>(0.675) |
| --- | --- | --- | --- | --- | --- | --- |
| SWE<5 | 2(3.8) | 15(28.3) | 5(9.4) | 0 | 7(13.2) | 15(28.3) |
| 5<SWE<12 | 3(5.7) | 22(41.5) | 1(1.9) | 0 | 3(5.7) | 23(43.4) |
| SWE>12 | 0 | 2(3.8) | 3(5.7) | 0 | 2(3.8) | 3(5.7) |
| Elastography by TE (n=23) | FIB-4 (<1.3) | FIB-4 ( 1.3-3.25) | FIB-4 ( >1.3) | NFS<(-0.455) | NFS (-0.455-0.675) | NFS>(0.675) |
| TE<5 | 0 | 1(4.3) | 1(4.3) | 0 | 1(4.3) | 1(4.3) |
| 5<TE<12 | 2(8.7) | 14(60.9) | 0 | 0 | 4(17.4) | 12(52.2) |
| TE>12 | 1(4.3) | 3(13) | 1(4.3) | 0 | 1(4.3) | 4(17.4) |
Data are presented n (%).NFS; NAFLD score, SWE; shear wave elastography, TE; transient elastography
